# A Rational Conclusion

**Published:** 1886-06

**Authors:** 


### A Rational Conclusion.

The editor of the *Medical Record*, of May 15, in speaking of the organization of the Executive Committee of the Ninth International Medical Congress says: "But the organization and management are definitely settled, and we have no desire now to hamper its future work. We do not think that hereafter the International Medical Congress will meet with any aggressive opposition or criticism. Its managers have chosen their course, and we shall be glad, for the sake of our country's reputation, to see them successful in it." In another paragraph, the same editor says: "Still, we say to foreign delegates, you will meet a large number of able and hospitable gentlemen at the Congress, you will be made warmly welcome by all Americans, and you will hear no quarrelling while you are in the States."

Kindly sentiments and assurances of future peace, even though late in finding expression, are none the less welcome. And if the Executive Committee of the Congress is to be permitted to carry forward its responsible work without further opposition, "our country's reputation" will be abundantly vindicated by the result.—*Journal of the American Medical Association.*

